# A multicenter survey on toxoplasmosis knowledge among pregnant women in Poland (the TOWER study)

**DOI:** 10.1186/s12884-018-2031-7

**Published:** 2018-10-03

**Authors:** Jacek Smereka, Lukasz Szarpak, Kurt Ruetzler, Yehoshua Schacham, Adam Smereka, Marek Dabrowski, Marzena Terpilowska, Lukasz Terpilowski, Ishag Adam

**Affiliations:** 1 Department of Emergency Medical Service, Laboratory of Experimental Medicine and Innovative Technology, Wroclaw Medical University, Wroclaw, Poland; 2 Faculty of Medicine, Lazarski University, 43 Swieradowska Str., 02-662 Warsaw, Poland; 3 Department of Outcomes Research, Anesthesiology Institute, Cleveland Clinic, Cleveland, OH USA; 4 Department of Clinic of Gastroenterology and Hepatology, Wroclaw Medical University, Wroclaw, Poland; 5 Chair and Department of Medical Education, Poznań University of Medical Sciences, Poznan, Poland; 6 Division of Neonatology, Department of Pediatrics, Faculty of Health Sciences, Wroclaw Medical University, Wroclaw, Poland; 7 Faculty of Medicine, University of Khartoum, Khartoum, Sudan

**Keywords:** Toxoplasmosis, Pregnancy, Toxoplasma gondii, Awareness

## Abstract

**Background:**

The seroprevalence of *Toxoplasma gondii* ranges widely in different areas of the world and different populations. Although toxoplasmosis is typically benign and asymptomatic, it induces major complications in immunocompromised individuals and during pregnancy. Prevention of maternal primary infection constitutes the major tool for avoiding congenital *T. gondii* infections and toxoplasmosis complications. The preventive measures depend on the women’s knowledge about toxoplasmosis. The aim of the study was to assess the knowledge on toxoplasmosis among pregnant women in Poland.

**Methods:**

The study was conducted between October 2016 and January 2017 in 3 Polish cities. During a visit in a hospital outpatient clinic, pregnant women aged > 16 years fulfilled a previously validated questionnaire. The questions concerned personal data (age, parity, educational level, place of residence), toxoplasmosis knowledge (etiology, routes of transmission, symptoms, sequelae), and sources consulted to collect information.

**Results:**

Overall, 465 pregnant women participated in the survey; 439 (94.4%) were aware of toxoplasmosis. Toxoplasmosis was perceived as a zoonotic disease by 77.4%, as a parasitic disease by 41.7%, as a disease transmitted through poor hand hygiene by 8.6%, as a childhood illness by 4%, and as a congenital disease by 0.4%. Regarding the transmission route, 84.5% of women pointed at a domestic cat, 46.7% at eating raw or undercooked meat. The total of 84.3% did not know toxoplasmosis symptoms, and 12.0% stated that they did not present the symptoms. In multivariate analysis, younger age (OR, 2.74; 95% CI, 1.67–4.49; *p* <  0.001), city residence (OR, 13.45; 95% CI, 3.12–57.89; *p* <  0.003), and higher education level (OR, 6.81; 95% CI, 3.69–12.59; *p* <  0.001) were significantly associated with better knowledge of toxoplasmosis, and the number of children (OR, 0.32; 95% CI, 0.22–0.48; *p* <  0.001) – with higher knowledge of the symptoms.

**Conclusions:**

Among pregnant women in Poland, the basic knowledge on toxoplasmosis is very high (94.4%). Younger age, city residence, higher education level, and the number of children turned out significantly associated with better knowledge of *T. gondii* and toxoplasmosis symptoms.

**Electronic supplementary material:**

The online version of this article (10.1186/s12884-018-2031-7) contains supplementary material, which is available to authorized users.

## Background

*Toxoplasma gondii* is an obligate intracellular protozoan parasite transmitted to humans through ingestion of food containing infectious oocysts that had been contaminated by feline feces (the definitive host), or ingestion of tissue cysts in undercooked meat of intermediate hosts, e.g. pork or lamb [1–3]*.* The seroprevalence of *T. gondii* ranges widely in different areas of the world and different populations, from ≤10% to almost 100% [4, 5]. Although toxoplasmosis is typically benign and asymptomatic, it induces major complications in immunocompromised individuals and during pregnancy, where it can lead to miscarriage and congenital disease [6]. Congenital toxoplasmosis is a substantial burden for public health worldwide [7]. A previous report showed that about 41% and 5% of Polish pregnant women had specific IgG and IgM antibodies, respectively, and estimated that 1.5/1000 neonates were infected in utero [8].

Since there is no vaccine against *T. gondii*, prevention of maternal primary infection constitutes the major tool for avoiding congenital *T. gondii* infections and their complications. The preventive measures mostly depend on the women’s knowledge about toxoplasmosis, its transmission, and origin. Previous studies have shown different levels of pregnant women’s knowledge regarding the risk and consequences of toxoplasmosis infection during pregnancy. It remains controversial, however, if improved knowledge and access to reliable information about sources of infection are suitable to consequently change women’s behavior during pregnancy [9, 10]. Nevertheless, the aim of the study was to assess the toxoplasmosis knowledge of pregnant women in Poland.

## Methods

The study was approved by the Institutional Review Board of the Polish Society of Disaster Medicine (approval No. 14.09.2016.IRB) and by the administrations of the healthcare institutions participating in the study. All data were anonymously collected and safely stored.

This cross-sectional, multicenter study was conducted between October 2016 and January 2017 in third-level hospitals in 3 Polish cities: Wroclaw, Warsaw, and Poznan. Pregnant women aged above 16 years who had a regular antenatal clinic visit in an outpatient clinic were asked to participate in the study. Once a patient agreed, written consent was obtained and the patient was interviewed in accordance with the pre-defined questionnaire by 2 of the researchers.

### Questionnaire

The questionnaire was previously prepared by 2 researchers and validated in a pilot study conducted in September 2016 in Wroclaw, Poland. The comments of the participating pregnant women were used to improve the wording of some items in the questionnaire and eliminate redundant questions. The pilot study also allowed to determine the time needed to complete the questionnaire.

The questions addressed the following issues: personal data (age, parity, educational level, place of residence), knowledge about *T. gondii* (etiology, routes of transmission, symptoms, and sequelae), and sources consulted to collect information (Additional file 1).

Only closed-ended questions were used: dichotomous (e.g. gender, presence of children), nominal (e.g. educational level, marital status, knowledge of items) or multiple choice (e.g. sources of information).

### Statistical analysis

Statistical analysis was performed with the Statistica version 13.1EN software (StatSoft, Tulsa, OK, USA). Descriptive statistics were used to summarize the demographic variables. Demographics were categorized by gender, age (≤ 19, 20–25, 26–35, 36–45, > 45 years), education (did not graduate from middle school, graduated from middle school, graduated from secondary school, university degree), place of residence (city, village), and number of pregnancies (1, 2, ≥ 3).

The differences between the groups were analyzed with chi-square tests for categorical data and either an independent *t*-test or Mann-Whitney U test for continuous data. Pearson’s correlation coefficient was used to evaluate the correlations between variables. Linear regression was performed with the stepwise method to identify the variables most likely to predict a high level of knowledge regarding *T. gondii* among the pregnant women. The Durbin-Watson test was applied to identify an auto-correction in the regression analysis in which a value close to 2 indicated no first-order serial correlation. Multivariate logistic regression analyses allowed to identify factors associated with toxoplasmosis knowledge. Variables with *p* <  0.2 in univariate analyses were included in the multivariate analysis.

The *p*-value of < 0.05 was regarded as statistically significant.

## Results

The total of 500 pregnant women were asked to participate in the survey. Of those, 465 agreed, which resulted in the participation rate of 93%. The demographic data of all the 465 subjects are shown in Table 1.

**Table 1:** Demographic characteristics of pregnant women participating in the survey

Characteristics	*N* = 465
Age (years):	
≤ 19 years	25 (5.4%)
20–25 years	105 (22.6%)
26–30 years	137 (29.5%)
31–35 years	113 (24.3%)
36–40 years	83 (17.8%)
> 40 years	2 (0.4%)
Educational level	
did not graduate from middle school	53 (11.4%)
graduated from middle school	111 (23.9%)
graduated from secondary school	249 (53.5%)
university degree	52 (11.2%)
Place of residence	
Urban area	231 (49.7%)
Village	234 (50.3%)
Number of pregnancies:	
1	126 (27.1%)
2	218 (46.9%)
≥ 3	121 (26.0%)

The education level of the study participants varied - 53 (11.4%) did not graduate from middle school, 111 (23.9%) graduated from middle school, 249 (53.5%) graduated from secondary school and 52 (11.2%) had university degree. Near half of the study participants - 231 (49.7%) reside in urban area and 234 (50.3%) in rural area. Number of pregnancies of study participants varied – 1 pregnancy was reported in 126 study participants (27.1%), 2 in 218 (46.9%) and 3 and more in 121 (26.0%).

Overall, 439 (94.4%) of the 465 included pregnant women were aware of the existence of toxoplasmosis. Among the most common sources providing information about toxoplasmosis, the following were reported: medical doctors (76.1%), the Internet (45.6%), television (41.3%), books (12.0%), and mother, family, or friends (1.9%).

According to 77.4% of the pregnant women, toxoplasmosis was a zoonotic disease, 41.7% believed that it was a parasitic disease, 8.6% were convinced that toxoplasmosis was transmitted through poor hand hygiene, 4% maintained that toxoplasmosis was a childhood illness, and 0.4% stated that it was a congenital disease.

Regarding the question about the transmission route of *T. gondii*, 84.5% of women responded that it was transmitted via a domestic cat, and 46.7% pointed at eating raw or undercooked meat. Only 0.6% of the participants thought the parasite was spread through insect bites and 5.2% did not have knowledge on the subject.

The total of 84.3% of the surveyed did not know toxoplasmosis symptoms, and 12.0% stated that they did not present any symptoms of the disease. The pregnant women reported that the symptoms of toxoplasmosis included enlarged lymph nodes (3.4%), diarrhea (1.3%), and constipation (0.4%).

Among the preventative measures against toxoplasmosis, the respondents most frequently enumerated: avoiding contact with cats (83.9%), personal hygiene (46.0%), avoiding consumption of raw meat (45.8%), avoiding sandy beaches (4.9%), and avoiding contact with previously infected patients (0.4%).

In general, 83.2% of the participants believed that toxoplasmosis might be dangerous during pregnancy and according to 49.9% infection with the parasite could cause premature labor or miscarriage. When asked whether toxoplasmosis was a cause of any developmental defects in children (epilepsy, hypopigmentation, cataract, hydrocephalus, or heart defects), the vast majority of respondents (84.7%) reported no knowledge; 13.1% indicated heart defects as a potential consequence of toxoplasmosis, 11.4% pointed at hydrocephalus, 3.9% at epilepsy, and 1.3% at smallpox.

In the studied group, 83% did not know which trimester of pregnancy was associated with the greatest fetal risk related to the disease, whereas 17% indicated the first trimester.

Out of all the participants, 93.3% admitted that more emphasis should be put on health education related to toxoplasmosis prevention, 100% said that physicians should pay more attention to women at risk; 60.3% believed that the issue should be addressed in childbirth classes; 17.2% maintained that it should also be discussed by nutritionists.

To identify potential associations between high knowledge of *T. gondii* and socio-demographic characteristics, multivariate logistic regression analysis was conducted (Table 2). Younger age (OR, 2.74; 95% CI, 1.67–4.49; *p* <  0.001), city residence (OR, 13.45; 95% CI, 3.12–57.89; *p* <  0.003), and higher education level (OR, 6.81; 95% CI, 3.69–12.59; *p* <  0.001) turned out significantly associated with better knowledge of toxoplasmosis, and the number of children (OR, 0.32; 95% CI, 0.22–0.48; *p* <  0.001) – with higher knowledge of the symptoms.

**Table 2:** Results of the logistic regression

	OR	95% CI	*p*
Have you ever heard of toxoplasmosis? (YES)			
Advanced age	2.74	1.67–4.49	< 0.001
Place of residence, Urban area	13.45	3.12–57.89	< 0.001
Higher education level	6.81	3.69–12.59	< 0.001
Higher number of pregnancies	1.33	0.77–2.32	0.305
Do you know what symptoms are associated with toxoplasmosis? (YES)			
Advanced age	1.29	0.92–1.80	0.140
Place of residence, Urban area	4.75	2.60–8.67	< 0.001
Higher education level	5.97	3.65–9.77	< 0.001
Higher number of pregnancies	0.32	0.22–0.48	< 0.001
Do you think that toxoplasmosis can be dangerous to the fetus? (YES)			
Advanced age	1.46	1.07–1.99	0.015
Place of residence, Urban area	8.28	4.24–16.20	< 0.001
Higher education level	3.20	2.33–4.40	< 0.001
Higher number of pregnancies	0.75	0.54–1.05	0.095
Do you think that toxoplasmosis can cause premature labor or miscarriage? (YES)			
Advanced age	1.07	0.85–1.36	0.556
Place of residence, Urban area	3.50	2.39–5.13	< 0.001
Higher education level	2.14	1.66–2.74	< 0.001
Higher number of pregnancies	0.52	0.40–0.67	< 0.001
Do you think that infection with toxoplasmosis can cause one of the following developmental defects in the child? (KNOWS)			
Advanced age	1.05	0.75–1.46	0.779
Place of residence, Urban area	6.90	3.51–13.54	< 0.001
Higher education level	6.98	4.16–11.72	< 0.001
Higher number of pregnancies	0.26	0.17–0.40	< 0.001
In which trimester of pregnancy do you think the fetus is most at risk for toxoplasmosis? (KNOWS)			
Advanced age	1.05	0.76–1.44	0.763
Place of residence, Urban area	4.17	2.37–7.33	< 0.001
Higher education level	12.5	6.91–22.69	< 0.001
Higher number of pregnancies	0.24	0.16–0.37	< 0.001

## Discussion

The main finding of the current study was a high (94.4%) basic knowledge on toxoplasmosis among the studied pregnant women in Poland. This correlates with the recent results that pregnant women in Geneva, Switzerland had a high (87%) knowledge about toxoplasmosis [11], and about 75.3% of Dutch women had been exposed to information concerning toxoplasmosis [12]. These outcomes contrast with previous worldwide findings reporting a generally low level of knowledge about toxoplasmosis, risk factors, prevention, and consequences of the disease among pregnant women [13]. Ogunmodede et al. reported that less than half of pregnant women in the USA had heard about toxoplasmosis, and that higher education levels were significantly associated with knowledge of the disease [14]. In Sri Lanka, *T. gondii* awareness among pregnant women equaled only 4.4%. Health personnel and media were the sources of information in 46.2% and 53.8%, respectively [15]. Likewise, it was reported that 3/4 of 400 pregnant Saudi women had never heard of toxoplasmosis, and those who lacked that knowledge were at 4.04 times higher risk of toxoplasmosis [16]. Interestingly, only 27.8% of the studied pregnant women in Niterói, Rio de Janeiro claimed to know about toxoplasmosis. Those who were conscious of *T. gondii* had a significantly lower probability of being seropositive to *T. gondii* IgG [17].

Previous studies reported that health education of pregnant women might help reduce the risk of congenital toxoplasmosis [10, 18]. Another study performed in Belgium indicated that health education led to a 63% reduction in T. gondii seroconversion [19]. Toxoplasmosis-related ducation of pregnant women in Poland increased their knowledge about the disease and its prevention twice in a 4-year period [20].

The total of 2598 pregnant women from Malaysia, Philippines, and Thailand were randomly surveyed in order to determine their knowledge and practices regarding *T*. *gondii* infection. Only 11% of them had read, heard, or seen information regarding toxoplasmosis and only 3.5% were aware of having been tested for the infection [21].

The practical implication of our study is the necessity to improve the knowledge of *T. gondii* and toxoplasmosis symptoms in pregnant women especially in rural areas or with lower education level.

The limitation of our study is the use of close questions in the questionnaire the free-form response was not allowed. Our study included only participants from several centers and certain geographic area and it may not reflect the whole country. The strength of our study is a high number of participants and the fact that it was to our best knowledge the first study on knowledge on toxoplasmosis among the pregnant women in Poland.

## Conclusions

Among pregnant women in Poland, the basic knowledge on toxoplasmosis is very high (94.4%). Younger age, city residence, higher education level, and the number of children turned out significantly associated with better knowledge of *T. gondii* and toxoplasmosis symptoms.

## Additional file

Additional file 1: Questionnaire. (DOCX 18 kb)

## Abbreviations

CI: Confidence interval
OR: Odds ratio
*T.gondii*: *Toxoplasma gondii*
