# Supplementary material for: A multicenter survey on toxoplasmosis knowledge among pregnant women in Poland (the TOWER study)
Source: BMC Pregnancy Childbirth. 2018 Oct 3;18:389. doi: 10.1186/s12884-018-2031-7 (PMC6171245; doi:10.1186/s12884-018-2031-7)
Supplement: Supplementary file 1 — Questionnaire. (DOCX 18 kb) [file 12884_2018_2031_MOESM1_ESM.docx]

**The knowledge on toxoplasmosis**

**The questionnaire is addressed to pregnant women and concerns their knowledge about *Toxoplasma gondii* infections.**

1. **Age**

**………………………..** years

1. **Education level**

- primary school
- middle school
- secondary school
- university education

1. **Place of residence**

- rural area
- village

1. **Which child do you expect?**

- 1
- 2
- 3 and more

1. **Have you ever heard of toxoplasmosis?**

- yes
- no

1. **If you have heard of toxoplasmosis, how did you learn about it?**

- Internet
- TV
- mother, family or friends
- doctor
- book
- other, what? ………………………………………………………………………………………….

1. **What do you think toxoplasmosis is (you can choose from several answers)?**

- a childhood disease
- a zoonosis
- a dirty hands disease
- a parasitic disease
- a viral disease
- a congenital disease

1. **Do you know how toxoplasmosis can be transmitted? (several answers can be chosen)**

- by the domestic cat
- by consuming raw or undercooked meat
- by insect bites
- due to the passage of the parasite through the placenta.
- don't know

1. **Do you know what symptoms are associated with toxoplasmosis? (several answers can be chosen)**

- there are no symptoms of this disease
- swollen lymph nodes
- eyeball inflammation
- diarrhoea
- constipations
- don't know

1. **Do you know how to protect yourself against toxoplasmosis?**

- avoid contact with cats
- not to consume raw meat
- avoid infected persons
- take care of personal hygiene, wash hands often
- avoid sandboxes
- other, what?.....................................................................................................................

1. **Do you think that toxoplasmosis can be dangerous to the foetus?**

- yes
- no
- don't know

1. **Do you think that toxoplasmosis can cause premature labour or miscarriage?**

- yes
- no
- don't know

1. **Do you think that infection with toxoplasmosis can cause one of the following developmental defects in the child? (you can choose from several answers)**

- Epilepsy
- Microcephalus
- Cataract
- Hydrocephalus
- heart defects
- don't know

1. **During pregnancy, in your opinion, in which trimester of pregnancy the foetus is most at risk for toxoplasmosis?**

- in the first trimester
- in the second trimester
- in the third trimester
- don't know

1. **Do you think that more emphasis should be placed on health education on this subject in hospitals, birthing schools, etc.?**

- yes
- no
- don't know

1. **How would you like to promote knowledge about toxoplasmosis? (you can select more answers or express your own opinion)**

- doctors should pay more attention to raising awareness among women
- this should be mentioned in birth schools
- this issue should be brought to the attention of women, including nutritionists
- other, what? ……………………………………....................…………………………………………
